# The Psychic Life of Micro-Organisms

**Published:** 1889-04

**Authors:** 


THE PSYCHIC LIFE OF MICRO-ORGANISMS. A STUDY IN EXPERIMENTAL PSYCHOLOGY. BY ALFRED BINET. Translated from the French by Thomas McCormack, with a preface by the author written especially for the American edition. Chicago; 1889. The Open Court Publishing Company. Cloth, 75 cents. Paper, 50 cents.

M. Alfred Binet, the collaborator of Ribot and Fèrè, and one of the most eminent representatives of the French School of Psychology, has presented in the above work the most important results of recent investigations into the world of Micro-Organisms. The subjectis a branch of comparative psychology little known; as the data of this department of natural science lie scattered for the most part in isolated reports and publications, and no attempt has hitherto been made to collate and present them in a systematized form.

